# Testing a Self-Compassion Intervention Among Job Seekers: Self-Compassion Beneficially Impacts Affect Through Reduced Self-Criticism

**DOI:** 10.3389/fpsyg.2020.01371

**Published:** 2020-07-02

**Authors:** Loes M. Kreemers, Edwin A. J. van Hooft, Annelies E. M. van Vianen, Sophie C. M. Sisouw de Zilwa

**Affiliations:** ^1^Research Group Psychology for Sustainable Cities, Amsterdam Research Institute for Societal Innovation, Amsterdam University of Applied Sciences, Amsterdam, Netherlands; ^2^Work and Organizational Psychology, University of Amsterdam, Amsterdam, Netherlands; ^3^Erasmus Happiness Economics Research Organization, Erasmus University Rotterdam, Rotterdam, Netherlands

**Keywords:** intervention, job search, negative affect, positive affect, self-compassion, self-criticism

## Abstract

Job search is associated with various obstacles and difficulties that can elicit negative emotions and undermine positive emotions. Having self-compassion may benefit job seekers’ well-being by stimulating more balanced emotional responses to negative job search experiences. In an intervention study we examined whether state self-compassion can be increased among job seekers through writing exercises in which job seekers are instructed to reflect with self-compassion on their negative job search experiences. We further examined whether the self-compassion intervention benefited job seekers’ affective responses, through reducing self-criticism. We designed a between-participants field experiment with two conditions (i.e., self-compassion vs. control) and three measurement times 1 week apart: a baseline questionnaire, the intervention with a second questionnaire, and a follow-up questionnaire (*N* = 180). Results show that the self-compassion writing exercise increased job seekers’ state self-compassion, which in turn related to their affective responses to job search. Specifically, their negative deactivating affect (e.g., sadness) was lower and their positive deactivating affect (e.g., calmness) was higher immediately after the self-compassion writing exercise than after reflecting freely (i.e., the control condition). The effects on job seekers’ affect were partially mediated by reduced self-criticism.

## Introduction

Most people search for a job at some stage in their career, for example when entering the labor market after finishing school, when a temporary contract ends, after being laid off, or when desiring to make a career move. The chance of finding employment increases to the extent that job seekers put effort in their search and search intensively (Kanfer et al., 2001; Van Hooft et al., in press). However, job search can be difficult and stressful, and is associated with various negative experiences such as rejections or failing to find job leads (Song et al., 2009), making job seekers prone to feelings of self-doubt, anxiety, self-criticism, and rumination (e.g., Wanberg et al., 2012). This makes job search an emotional experience that may harm job seekers’ well-being (McKee-Ryan et al., 2005). Understandably, job seekers can become discouraged during their search and postpone or hold off searching for a job, which lengthens the duration of their unemployment, leading to adverse consequences for themselves, their families, and society as a whole (Klehe and Van Hooft, 2018).

Several scholars have called for research that investigates ways in which job seekers can deal with negative job search experiences to reduce their negative emotional impact (e.g., Song et al., 2009; Turban et al., 2013). This is important because while negative affect during job search frequently occurs, it is positive affectivity that enhances job search success (Turban et al., 2013). Self-compassion may be a promising coping mechanism for job seekers when experiencing negative job search events because self-compassioned cognitions make individuals respond to failure with kindness to the self, make them aware that failure is human, and let them acknowledge their emotions with a healthy distance (Neff, 2003a). A recent correlational study has indeed shown that job seekers higher on trait self-compassion were less affected by negative job search experiences, such that they felt more positively and less negatively while searching for a job (Kreemers et al., 2018).

Although it is promising that naturally occurring variation in self-compassion is related to job seeker affect, an important remaining question is if and how job seekers’ self-compassion can be increased, and whether this results in more emotionally balanced responses during job search. In the current study, we therefore build on previous research (e.g., Leary et al., 2007; Shapira and Mongrain, 2010; Breines and Chen, 2012; Zhang and Chen, 2016) to develop an intervention composed of online writing exercises that facilitate taking a self-compassioned perspective toward job search difficulties. In an experimental field study we tested whether this online intervention indeed increases job seekers’ state self-compassion and subsequently makes them feel better, as compared to a control condition composed of online writing exercises in which job seekers reflect freely on their difficulties.

The current study aims to contribute to job search interventions research, by showing that self-compassion has the potential to aid job seekers in effectively dealing with stress that occurs as a consequence of negative job search experiences. This is important as research thus far could not identify stress management interventions as effective components of job search interventions (Liu et al., 2014). Further, we extend prior research by Kreemers et al. (2018), who showed a relationship between trait self-compassion and job seekers’ affect. Because their design was correlational, we cannot rule out the possibility that this relationship is spurious (e.g., affected by omitted third variables). Our experimental design allows for drawing causal conclusions regarding the impact of self-compassion on job seekers’ affective responses to their job search experiences. This is important for theory, as it provides a stringent test of self-compassion theory in the context of job search, but also for practice. Practical recommendations often rely on the notion of causality (e.g., Aguinis and Edwards, 2014; Eden, 2017). Hence, this study provides tools that can be implemented in practice, such as writing exercises to facilitate taking a self-compassioned perspective on job search difficulties to benefit job seekers’ emotions.

In addition to testing whether writing exercises promote job seekers’ state self-compassion and consequently positively influence their affect, we aim to increase our understanding of the process through which self-compassion influences affect. Based on theorizing and prior research, we propose reduced self-criticism as underlying mechanism that drives the effects of the self-compassion intervention. Although previous research has shown a negative relation between self-compassion and self-criticism (Gilbert and Procter, 2006), self-criticism has not been tested as mediator between self-compassion and affect. As such, we aim to contribute to the self-compassion literature by uncovering an important underlying mechanism that explains the effects of self-compassion. Understanding how self-compassion makes job seekers feel better is also important for practice to target sensitive groups (e.g., those who suffer more from self-criticism; Shapira and Mongrain, 2010) who could benefit from a self-compassion intervention.

### Job Search and Mental Well-Being

Job search requests cyclical self-regulatory behavior that is purposive and self-organized (Kanfer et al., 2001). It includes a range of activities such as searching for vacancies, having network conversations, contacting employment agencies, and submitting applications. The amount of time and effort that job seekers spend on job search activities is positively related to job search success (Kanfer et al., 2001; Van Hooft et al., in press). However, during job search there is hardly a clear pathway toward reaching the desired employment outcome. People oftentimes receive little feedback on the steps they take along the way other than rejections, which makes it hard to perceive progress. Results of a daily diary study show that a lack of job search progress adversely relates to how job seekers feel (Wanberg et al., 2010). Furthermore, the more time job seekers spend on their job search, the more negative job search experiences they have and the higher their distress (Song et al., 2009). Qualitative research has indicated that job seekers are especially prone to feelings of self-doubt, anxiety, self-criticism, and rumination (Wanberg et al., 2012). Job search difficulties such as failure to find suitable leads, lack of job search progress, and rejections elicit negative thoughts and feelings, which in turn harm job seekers’ mental well-being. In addition to eliciting negative emotions, job search difficulties also undermine positive emotions (Kreemers et al., 2018), which has consequences for job search outcomes, as especially positive feelings are related to job search success (Turban et al., 2013). Therefore, it is important to identify how negative affective responses during job search can be reduced and positive affective responses can be increased.

### Self-Compassion

A healthy way to respond to negative experiences such as setbacks, humiliation, and failure is reflecting on them with self-compassion. Self-compassion emanated from Buddhist philosophy and is an emotion-focused coping strategy. It is different from traditional emotion regulation strategies in that it does not aim to alter people’s emotional state, but rather allows for emotions to exist in kind awareness (Neff, 2003a). Self-compassion entails three basic components: self-kindness, common humanity, and mindfulness. People who are self-compassioned respond to their failures with self-kindness and understanding (self-kindness) rather than with harsh self-judgment, they are understanding of the shared nature of their experiences (common humanity) rather than feeling isolated, and they acknowledge their emotions with mindful acceptance (mindfulness) rather than ignoring or exaggerating them (Neff, 2003b). While these components are conceptually distinct, they also interact so as to mutually enhance and engender one another (Neff, 2003a). Self-compassion should not be confused with self-pity because people who pity themselves feel isolated in their misery and fail to recognize the shared nature of their experience (Neff, 2003a). Self-compassion also differs from self-esteem, in that it constitutes more stable feelings of self-worth enabling people to see themselves and their flaws with more clarity than self-esteem, which is more contingent on positive ego-focused outcomes (Neff and Vonk, 2009).

Self-compassionate individuals are better at withstanding experiences of pain and failure, because these experiences are not met with harsh self-condemnation, feelings of isolation, or over-identification with thoughts and emotions. Therefore, there are lower incidences of anxiety and depression among self-compassionate individuals and better mental health outcomes such as more connectedness and subjective well-being (Neff, 2003a, b; Neff et al., 2005, 2007). Previous research shows that people who are able to reflect on their experiences with self-compassion generally feel better and have more moderate responses to unpleasant and self-relevant experiences than people who are not able to reflect on their experiences with self-compassion (e.g., Neff et al., 2005, 2007; Leary et al., 2007). For example, research showed that students who were instructed to be more self-compassioned experienced less negative affect in response to unfavorable feedback (Leary et al., 2007). Other research showed that students dealt more adaptively with academic failure when they had more self-compassion (Neff et al., 2005). The beneficial role of self-compassion is also apparent in studies with clinical samples with participants suffering from shame or trauma (e.g., Thompson and Waltz, 2008; Raes, 2010).

We propose that self-compassion can be helpful during the job search process, which is usually full of self-relevant negative experiences. A recent correlational study indeed showed that job seekers higher on trait self-compassion experienced more positive and fewer negative emotions during their job search than job seekers lower on trait self-compassion (Kreemers et al., 2018). Given the aforementioned theorizing and empirical support for the beneficial psychological effect of self-compassion in times of perceived failure and setback, we expect that stimulating job seekers to reflect on their job search experiences with self-compassion will decrease negative affect and increase positive affect in comparison to job seekers who reflect on their experiences freely.

### Enhancing State Self-Compassion

Given that state self-compassion relates to an array of beneficial psychological outcomes, there have been various attempts to develop methods aimed at stimulating self-compassion (e.g., Gilbert and Procter, 2006; Adams and Leary, 2007; Leary et al., 2007; Shapiro et al., 2007; Shapira and Mongrain, 2010; Neff, 2011; Breines and Chen, 2012; Smeets et al., 2014; Zhang and Chen, 2016; Au et al., 2017). The most simple example is letting participants know that they were not alone in their experience and encouraging them to not be hard on themselves (cf. Adams and Leary, 2007; Breines and Chen, 2012, Experiment 3). More elaborate forms of stimulating self-compassion are continuous practice with a multitude of exercises intended to move toward a kinder, more mindful mindset that acknowledges the shared nature of negative experiences (see Neff, 2011; Smeets et al., 2014). There are also various therapy forms that have incorporated self-compassion (e.g., Compassion-Based Therapy, Gilbert and Procter, 2006; Mindfulness-Based Stress Reduction, Kabat-Zinn, 1982) and have been shown to successfully increase self-compassion (e.g., Shapiro et al., 2007; Au et al., 2017). A thorough but more accessible option than weekly meetings and therapy is having participants engage in self-compassion writing exercises (Leary et al., 2007; Breines and Chen, 2012, Experiment 5; Shapira and Mongrain, 2010; Zhang and Chen, 2016). Participants who were instructed to reflect on a personal weakness and take a self-compassioned and understanding perspective have been shown to have more state self-compassion than participants who were instructed to contrast their weakness with things they were proud of or describe their hobby after reflecting on a weakness (Breines and Chen, 2012).

Based on these positive effects in previous studies (e.g., Leary et al., 2007; Breines and Chen, 2012, Experiment 5; Zhang and Chen, 2016), in the current study we adopted the self-compassion writing exercise method and adapted it to the job search setting. Specifically, we instructed job seekers online to reflect on their worst job search experience and then asked them to reflect on this experience with self-compassion (i.e., self-compassion condition) or freely (i.e., control condition). Based on previous research using a comparable design (Leary et al., 2007, Experiment 5), we expect that after the writing exercise job seekers in the self-compassion condition will have more state self-compassion than job seekers in the control condition, which in turn will result in less negative affect and more positive affect. Previous research by Shapira and Mongrain (2010), who also made use of writing exercises, has shown that the effects of self-compassion interventions can have lasting effects. Therefore, we measured job seekers’ affect immediately after the exercises and we measured affect in a follow-up measurement 1 week later. As such, we extend previous research by Leary et al. (2007), who measured affect only directly after the exercises, by exploring whether the effects of the self-compassion exercise last over time.

### The Mediating Role of Self-Criticism

Furthermore, we expect that the beneficial influence of state self-compassion on affect can be attributed to reduced self-criticism. Negative experiences in personally relevant areas of life, such as job search, can make people more self-critical (Wanberg et al., 2012; Pinto-Gouveia et al., 2013). When people perceive personal failure or inadequacies (e.g., during job search), they tend to have an exaggerated focus on the implications of this experience for their self-worth, leading to feelings of isolation and overly severe judgments and criticism of the self (Neff, 2003a). Indeed negative job search experiences such as rejections or lack of progress have been shown to make job seekers prone to feelings of self-criticism (Wanberg et al., 2012). Neff (2003a) emphasizes that self-compassion is most relevant in situations that elicit feelings of shame and self-criticism, because self-compassion counteracts these feelings by means of its three mutually enhancing components. Self-kindness softens the self-consciousness that is strengthened though harsh self-judgment. Common humanity, that is, realizing that failure and personal suffering is shared, lessens the blame placed on oneself, further reducing self-criticism (Neff, 2003a). Finally, mindfulness contributes to reducing self-criticism by increasing the other two components of self-compassion. The non-judgmental, detached stance of mindfulness increases self-understanding and self-kindness (Jopling, 2000), whereas the balanced perspective-taking of mindfulness directly counters the egocentrism that causes feelings of isolation and separateness from the rest of humanity and thus increases feelings of interconnectedness (Elkind, 1967).

Empirical evidence further supports the notion that self-compassion is negatively related to self-criticism (Neff, 2003b; Gilbert and Procter, 2006; Neff et al., 2007), and that self-criticism is related to maladaptive outcomes such as depression (Blatt et al., 1982; Dunkley and Blankstein, 2000; Dunkley et al., 2003) and higher negative affect and lower positive affect (Zuroff et al., 1999). Therefore, we expect that reducing job seekers’ self-critical thoughts through self-compassioned cognitions when reflecting on a negative job search experience will promote their positive affect and reduce their negative affect. In other words, we expect that job seekers in the self-compassion condition report lower negative affect and higher positive affect than job seekers in the control condition as mediated by state self-compassion and self-criticism.

## Materials and Methods

### Participants and Design

We designed a between-participants experimental field study with two conditions and three measurement times. More specifically, we administered a baseline questionnaire before the intervention at Time 0, conducted the intervention (i.e., a self-compassion or control writing exercise) 6 days^1^ later, immediately followed by the Time 1 questionnaire, and a Time 2 questionnaire 5–7 days later.

Job search occurs in several stages of people’s lives, as reflected by job seeking research that has typically sampled students seeking for employment upon graduation, unemployed individuals looking for reemployment, and employed individuals seeking for a job change (e.g., Boswell et al., 2012; Van Hooft et al., in press). Rather than restricting to a particular group, in the present study we included job seekers from all these groups. Specifically, we recruited job seekers who were searching for a paid job (of at least 20 h) via the alumni department of a Dutch university, employment agencies, and social media, to participate in a study about job search. To be eligible for the study, participants had to be currently searching for a job and had to have searched in the last month. This ensured that participants had job search experiences to reflect on during the self-compassion/control writing exercise. Participants received €10 for completing all three questionnaires and the writing exercise.

A total of 354 participants started the Time 0 questionnaire. Of these, 288 participants met the eligibility criteria (currently searching for a job and having searched in the last month) and 205 finished the self-compassion/control writing exercise and subsequent Time 1 questionnaire. We excluded 18 participants from the analyses because they indicated to have found a job at Time 1. We further read all responses to the writing exercises, which led to the exclusion of seven participants from the analyses because their responses were absent or unrelated to job search. The final sample consisted of 180 participants, with complete data on both Time 0 and Time 1. Of these, 173 participants also completed the Time 2 questionnaire.

The average age in the final sample of 180 participants was 29 years (*SD* = 9.17) and 75.60% were women (*n* = 136). Most participants (59.40%) had a paid (student) job (*n* = 107), in which they worked an average of 26.08 h a week (*SD* = 11.78), and 9.40% had an unpaid job (*n* = 17), with an average of 11.53 h a week (*SD* = 8.64). Of the employed participants 32.20% worked under temporary employment, 13.90% had a permanent position, 11.10% was volunteer or intern, and 4.40% worked as freelancer. The sample was generally highly educated (74.40% university degree, 9.40% higher vocational education). Some participants (16.70%) were still studying but would graduate within 6 months (within 3.37 months on average). Mean job search duration at Time 0 was 5.71 months (*SD* = 6.06).

### Procedure

At Time 0 participants received an e-mail with a link to the baseline questionnaire. After having filled out the informed consent, participants had 14 days to finish the baseline questionnaire. Participants received reminders 3, 5, and 7 days after receiving a questionnaire if they had not finished the questionnaire. The Time 0 questionnaire contained questions about^2^ job search history, demographics, trait self-compassion, self-criticism, and affect.

One day after finishing Time 0, participants received a link to the writing exercise and Time 1 questionnaire. Participants were first asked to describe their worst job search experience in the past period.^3^ After having provided a description of the event, participants were randomly assigned to the self-compassion condition or the control condition. In the self-compassion condition participants were asked to reflect on their negative job search experience with self-compassion and to report these reflections in writing. In the control condition participants were asked to reflect on their negative job search experience by freely describing their naturally occurring thoughts and feelings. The writing exercise was followed by the Time 1 questionnaire, which included measures of state self-compassion, self-criticism, and affect. Five days after filling out the Time 1 questionnaire participants received the link to the Time 2 questionnaire, which included the same measures as at Time 1. Participants had 3 days to finish the Time 2 questionnaire and received reminders at days 2 and 3 if they had not finished the questionnaire.

### Self-Compassion and Control Assignments

The self-compassion intervention was based on the laboratory experiment of Leary et al. (2007) with psychology students, showing that self-compassion can be manipulated with a writing exercise using three questions tapping into the three self-compassion dimensions. Original materials were translated into Dutch and adjusted to the job search context and pilot tested to ensure that the instructions were clear.^4^ First, participants in both conditions were asked to describe a negative job search event in detail, how many weeks ago it occurred, what happened, who was present, and what had led to the event. Then, participants in the self-compassion condition answered three questions regarding the negative event tailored to the three dimensions of self-compassion (cf. Neff, 2003a; Leary et al., 2007). Specifically, they were asked to (a) indicate in what ways other people experience similar events (*common humanity*), (b) write a paragraph directed to themselves in a tone they would use for a friend (*self-kindness*), and (c) picture the emotions that they associated with the event as temporary states and describe them objectively and with mindfulness (*mindful acceptance*).

In the control condition, participants were asked to freely reflect on the negative job search event and describe their naturally occurring thoughts and feelings. We deliberately chose an active writing control condition as opposed to a passive control condition in which no task was assigned to the participants to ensure that we could attribute any effect of the self-compassion condition to self-compassion rather than to the mere process of reflecting on and writing about a certain event (cf. Leary et al., 2007). This is because writing about an emotional event is an intervention by itself, which has been shown to positively influence people’s affective state (Pennebaker et al., 1990).

### Measures

#### Trait Self-Compassion

Trait self-compassion was measured at Time 0 with the Self-Compassion Scale (Neff, 2003b; Neff and Vonk, 2009), which consists of 26 statements (e.g., “I’m tolerant to my own flaws and inadequacies” and “I try to see my failings as part of the human condition”). Participants indicated the extent to which they agreed with these statements on a 5-point scale ranging from *strongly disagree* (1) to *strongly agree* (5). Cronbach’s alpha was 0.94.

#### State Self-Compassion

At Time 1 after the intervention, participants were asked to indicate on a 7-point scale to what extent they agreed with three statements regarding their current self-compassion regarding their job search. We selected three^5^ statements from the Self-Compassion Scale of Neff (2003b) that each reflected one component of self-compassion (cf. Breines and Chen, 2012; Experiment 4) and adjusted the items to make them specific to job search (i.e., “When I look back at my job search experiences of the past month: I now treat myself kindly with respect of my job search experiences; I now try to have a balanced stance toward my job search experiences; I see my weaknesses in search for a job now as part of being human”; α = 0.71).

#### Self-Criticism

Self-criticism was measured at Time 0 and 1 with 10 items of the Self-Criticism Questionnaire (Brewin et al., 1992), adapted to the current job search context. Specifically, we adjusted the formulation such that the items apply to how self-critical participants feel considering their job search at the moment of measurement rather than generally (e.g., “I am very critical of myself in searching for a job”; “I blame myself for things that go wrong during job search”; “I find that I don’t live up to my own standards or ideals in my job search). Participants were asked to indicate their agreement with the statements on a 5-point scale ranging from *totally disagree* (1) to *totally agree* (5). Cronbach’s alpha was 0.87 at both measurement times.

#### Affect

Affective responses can be classified along hedonic tone (i.e., positive vs. negative) and activation level (i.e., activating vs. deactivating) (Feldman Barrett and Russell, 1998; Russell, 2003; Yik et al., 2011). This results in four types of affect: activating negative affect (e.g., nervous), activating positive affect (e.g., enthusiastic), deactivating negative affect (e.g., disappointed), and deactivating positive affect (e.g., at ease). Accordingly, we measured affect at Time 0, 1, and 2 with a selection of 16 emotions from the PANAS (Crawford and Henry, 2004) that clearly fell into the four affect categories of the emotion circumplex (Yik et al., 2011). Each affect category was measured with four emotions, that is, negative activating affect with nervous, stressed, frustrated, and jittery (α = 0.84–0.87), negative deactivating affect with sad, disappointed, down, and downcast (α = 0.89–0.91), positive activating affect with enthusiastic, cheerful, lively, and energetic (α = 0.94–0.96), and positive deactivating with at ease, calm, relaxed, and laid back (α = 0.94 at all times). Participants indicated the extent to which they felt the emotions when they considered their job search experiences of the last month on a 5-point scale ranging from *strongly disagree* (1) to *strongly agree* (5).

#### Demographics

Demographics were measured at Time 0. Specifically, age, gender, education (primary school, high school level 1, 2, or 3, intermediate vocational education, higher vocational education, or university degree), employment position (employed or unemployed), and job search duration (in months) were measured as control variables, as meta-analyses has shown their importance to the job search process (Kanfer et al., 2001).

With confirmatory factor analyses in Mplus 7.11 we tested a 6-factor model with all Time 1 variables (i.e., state self-compassion, self-criticism, and the four affect variables) against theoretical plausible alternative models (i.e., collapsing state self-compassion and self-criticism, collapsing the two negative and the two positive affect variables, collapsing all affect variables). The 6-factor model fit the data significantly better than all alternative models (all Δχ^2^ larger than 101.39; all *p*-values < 0.05; all ΔCFI > 0.03).

## Results

### Preliminary Analyses

To assess whether the participants in the self-compassion (*n* = 82) and control condition (*n* = 98) differed before the intervention, we compared participants’ demographics, employment status, job search duration, trait self-compassion, self-criticism, and affect at Time 0 between both conditions. Supporting the successfulness of the random assignment, there were no significant differences between conditions for age, *t*(174.38) = 1.70, *p* = 0.09, gender, χ^2^(1) = 1.32, *p* = 0.72, education, χ^2^(1) = 0.53, *p* = 0.47, employment status, χ^2^(1) = 1.68, *p* = 0.20, job search duration, *t*(178) = 0.65, *p* = 0.52, trait self-compassion, *t*(178) = −1.57, *p* = 0.12, self-criticism, *t*(156.50) = 0.67, *p* = 0.51, negative activating affect, *t*(178) = −0.54, *p* = 0.59, negative deactivating affect, *t*(178) = −0.38, *p* = 0.70, positive activating affect, *t*(178) = −0.66, *p* = 0.51, and positive deactivating affect, *t*(178) = −0.08, *p* = 0.94. Given the absence of significant *a priori* differences between the conditions there is no need to control for these Time 0 variables when testing the effectiveness of the intervention. Table 1 shows the correlations between all study variables.

**TABLE 1:** Means, standard deviations, and correlations among the study variables.

	*M*	*SD*	1	2	3	4	5	6	7	8	9	10	11	12	13	14	15	16	17	18	19	20	21
**Time 0**																							
(1) Age	29.13	9.17																					
(2) Gender^a^	1.76	0.43	–0.03																				
(3) Education^b^	0.84	0.37	−0.31**	–0.04																			
(4) Employment status^c^	0.59	0.49	−0.24**	0.11	0.07																		
(5) Job search duration^d^	5.71	6.06	0.63**	–0.03	−0.19*	0.01																	
(6) Trait self-compassion	3.08	0.64	0.20**	−0.20**	–0.04	–0.13	0.07																
(7) Self-criticism	2.81	0.86	−0.23**	0.01	0.21**	0.07	0.00	−0.58**															
(8) Negative activating affect	4.38	1.41	−0.29**	0.18*	0.20**	–0.02	–0.10	−0.38**	0.45**														
(9) Negative deactivating affect	3.79	1.57	−0.17*	0.18*	0.05	–0.10	–0.04	−0.47**	0.50**	0.69**													
(10) Positive activating affect	3.89	1.49	–0.08	–0.10	0.03	0.05	−0.20**	0.31**	−0.32**	−0.32**	−0.43**												
(11) Positive deactivating affect	3.63	1.41	0.08	−0.21**	–0.11	–0.04	–0.11	0.47**	−0.46**	−0.53**	−0.47**	0.67**											
**Time 1**																							
(12) Condition^*e*^	0.46	0.50	–0.12	0.03	–0.05	0.10	–0.05	0.12	–0.05	0.04	0.03	0.05	0.01										
(13) State self-compassion	4.92	1.00	0.03	–0.01	0.02	0.00	0.05	0.37**	−0.34**	−0.29**	−0.30**	0.33**	0.36**	0.16*									
(14) Self-criticism	2.87	0.85	−0.30**	0.09	0.17*	0.08	–0.06	−0.56**	0.78**	0.52**	0.55**	−0.26**	−0.44**	–0.05	−0.44**								
(15) Negative activating affect	4.03	1.47	−0.26**	0.10	0.25**	0.02	–0.14	−0.35**	0.48**	0.71**	0.53**	−0.36**	−0.51**	–0.14	−0.44**	0.58**							
(16) Negative deactivating affect	3.81	1.47	–0.10	0.18*	0.07	–0.02	–0.06	−0.39**	0.45**	0.47**	0.64**	−0.44**	−0.44**	–0.15	−0.52**	0.60**	0.73**						
(17) Positive activating affect	3.96	1.39	0.05	–0.10	–0.08	–0.05	–0.06	0.33**	−0.35**	−0.34**	−0.42**	0.57**	0.48**	0.09	0.48**	−0.35**	−0.47**	−0.59**					
(18) Positive deactivating affect	4.06	1.37	0.08	−0.18*	−0.15*	–0.04	0.04	0.38**	−0.40**	−0.52**	−0.48**	0.42**	0.57**	0.17*	0.48**	−0.45**	−0.69**	−0.64**	0.66**				
**Time 2**																							
(19) Negative activating affect	3.64	1.39	−0.27**	0.13	0.16*	0.00	–0.10	−0.37**	0.50**	0.70**	0.56**	−0.41**	−0.48**	–0.09	−0.39**	0.53**	0.78**	0.61**	−0.50**	−0.64**			
(20) Negative deactivating affect	3.18	1.46	–0.07	0.09	0.08	–0.05	0.06	−0.37**	0.40**	0.52**	0.61**	−0.40**	−0.43**	–0.09	−0.44**	0.44**	0.57**	0.66**	−0.50**	−0.50**	0.73**		
(21) Positive activating affect	4.22	1.44	0.08	–0.08	–0.04	–0.08	–0.08	0.23**	−0.30**	−0.27**	−0.27**	0.60**	0.45**	0.01	0.34**	−0.28**	−0.38**	−0.41**	0.68**	0.43**	−0.51**	−0.48**	
(22) Positive deactivating affect	4.29	1.40	0.24**	−0.17*	–0.12	0.00	0.09	0.39**	−0.47**	−0.48**	−0.43**	0.51**	0.55**	0.06	0.37**	−0.44**	−0.62**	−0.53**	0.54**	0.65**	−0.72**	−0.52**	0.64**

N = 180; ^*a*^1 = man, 2 = woman; ^*b*^0 = low or intermediately educated, 1 = highly educated; ^*c*^0 = no paid employment, 1 = paid employment; ^*d*^measured in months; ^*e*^0 = control condition, 1 = self-compassion condition. *p < 0.05, **p < 0.01.

### Effects of the Intervention

First, we tested the effects of the intervention on state self-compassion, measured directly after the writing exercises (Time 1). An independent *t*-test showed that participants in the self-compassion condition (*M* = 5.09, *SD* = 1.00) scored significantly higher on Time 1 state self-compassion than participants in the control condition (*M* = 4.78, *SD* = 0.99), *t*(178) = 2.11, *p* = 0.036, *d* = 0.32. In other words, the self-compassion writing exercises had a small to medium-sized positive effect on self-compassion toward job search experiences as compared to the control writing exercises.

**FIGURE 1:**
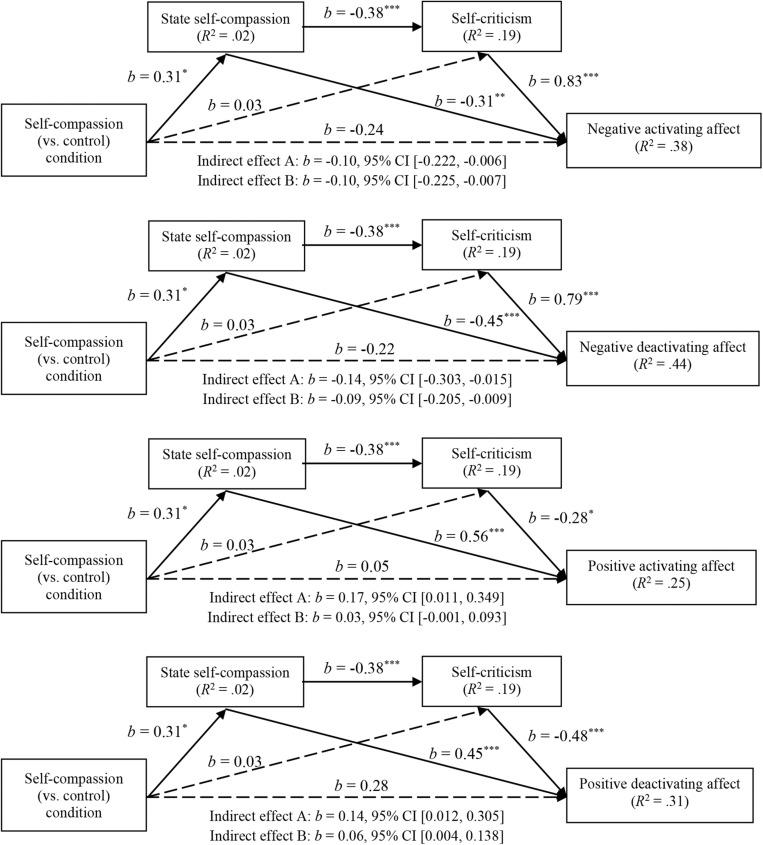
Results of the effects of condition on the four affect variables, as mediated by state self-compassion and self-criticism. Indirect effect A: condition through state self-compassion to affect. Indirect effect B: condition through state self-compassion and self-criticism to affect. The dashed lines are not significant. ^∗^*p* < 0.05, ^∗∗^*p* < 0.01, ^∗∗∗^*p* < 0.001.

Second, we tested the effects of the intervention on job seekers’ affect, as mediated by state self-compassion and self-criticism. We examined these indirect effects using the PROCESS bootstrapping method Model 6 (Hayes, 2012) in SPSS with the variables measured at Time 1. In line with affect theory, we ran an analysis for each of the four types of affect as dependent variable. In each analysis we entered condition as independent variable and state self-compassion and self-criticism as mediators. The pattern of results was roughly similar across different types of affect and is shown in Figure 1. For each type of affect there was no direct relation between condition and affect, *b* ranged from −0.24 to 0.28, *t*(176) ranged from −1.36 to 1.59, all *p-*values > 0.05, and no direct relation between condition and self-criticism, *b* = 0.03, *p* = 0.79. However, for each type of affect results did show two significant indirect effects: from condition through state self-compassion to affect (indirect effect A), and from condition to state self-compassion to self-criticism to affect (indirect effect B). This means that the effects of condition on affect run fully through state self-compassion, and partially also through self-criticism. As shown in Figure 1, the indirect effects are composed of the following relations. Regarding effect A, there was a positive relation between condition and state self-compassion. Further, state self-compassion had a negative relation with both types of negative affect and a positive relation with both types of positive affect. Regarding effect B, there was a negative relation between state self-compassion and self-criticism. Further, self-criticism had a positive relation with both types of negative affect and a negative relation with both types of positive affect.

Third, we examined the extent to which the effects of the intervention on job seeker affect hold over time from Time 1 to Time 2. Table 2 presents the raw means over time for the two conditions. We conducted four repeated measures analyses of variance (ANOVA) with the different types of affect at each measurement point (Times 0, 1, and 2) as within-participant variables and condition as between-participant variable. The repeated measures ANOVA assumptions of normality and homogeneity of variance were met. However, Mauchly’s test indicated that the assumption of sphericity had been violated for positive activating affect. Therefore, we corrected the degrees of freedom for positive activating affect using Huynh–Feldt estimates of sphericity (ε = 0.97). With contrast analyses we examined the changes in affect in the two conditions in more detail.

**TABLE 2:** Descriptives of the four affect variables at the Time 0, Time 1, and Time 2 measurement for the control and the self-compassion condition.

Affect	Condition	Baseline (T0)		Post intervention (T1)		Follow-up (T2)	
		*M*	*SD*	*M*	*SD*	*M*	*SD*
Negative activating affect	Control	4.31	1.40	4.19	1.42	3.75	1.33
	Self-compassion	4.40	1.44	3.76	1.50	3.51	1.44
Negative deactivating affect	Control	3.78	1.56	4.02	1.46	3.30	1.44
	Self-compassion	3.80	1.60	3.55	1.48	3.03	1.47
Positive activating affect	Control	3.82	1.48	3.85	1.39	4.21	1.40
	Self-compassion	4.00	1.51	4.13	1.35	4.23	1.51
Positive deactivating affect	Control	3.63	1.44	3.84	1.32	4.21	1.35
	Self-compassion	3.68	1.37	4.36	1.33	4.38	1.45

*N_*control*_ = 95; N_*self*__–compassion_ = 80.*

**FIGURE 2:**
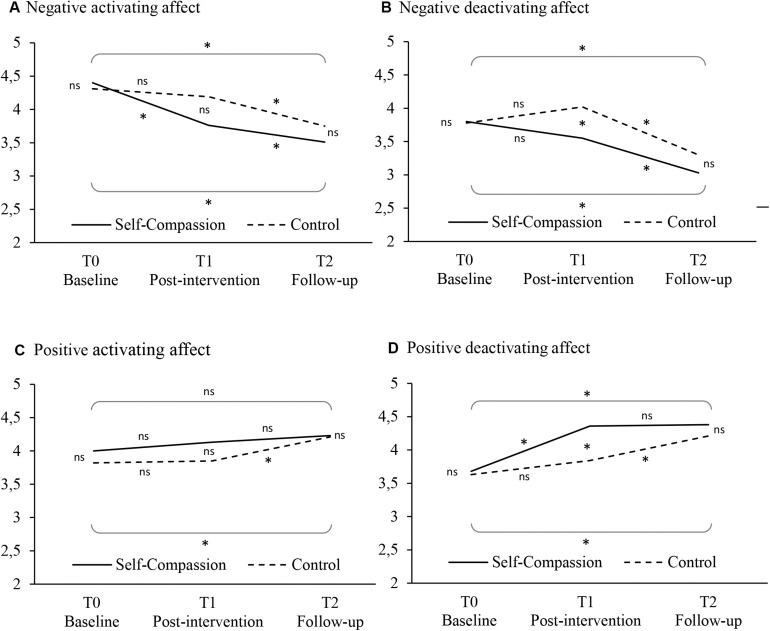
Graphical representation of the means of the four affect variables at the baseline, post-intervention, and follow-up measurements for the self-compassion and control condition. **(A)** Negative activating affect. **(B)** Negative deactivating affect. **(C)** Positive activating affect. **(D)** Positive deactivating affect. Significant difference in affect between times and conditions is indicated with an asterisk; non-significant differences is indicated with ns.

Results for negative activating affect showed a significant main effect of time, *F*(2,346) = 42.73, *p* < 0.001, $\eta_{\text{p}}^{2}$ = 0.20, and a significant interaction between time and condition, *F*(2,346) = 5.68, *p* = 0.004, $\eta_{\text{p}}^{2}$ = 0.03 (see Figure 2A). Contrast analyses showed a significant decrease in negative activating affect between Time 0 and Time 1 in the self-compassion condition (*M*_diff_ = −0.64, *p* < 0.001), but not in the control condition. At Time 1, participants’ mean negative activating affect was lower in the self-compassion condition than in the control condition, but this difference was only approaching significance (*M*_diff_ = −0.43, *p* = 0.055). Negative activating affect significantly decreased between Time 1 and Time 2 in both the self-compassion (*M*_diff_ = −0.25, *p* = 0.018) and the control condition (*M*_diff_ = −0.44, *p* < 0.001). There was no significant difference in negative activating affect between conditions at Time 2.

Similarly, for negative deactivating affect we found a significant main effect of time, *F*(2,346) = 27.28, *p* < 0.001, $\eta_{\text{p}}^{2}$ = 0.14, and a significant interaction between time and condition, *F*(2,346) = 3.24, *p* = 0.040, $\eta_{\text{p}}^{2}$ = 0.02 (see Figure 2B). Contrast analyses showed a decrease in negative deactivating affect between Time 0 and Time 1 in the self-compassion condition that approached significance (*M*_diff_ = −0.25, *p* = 0.082), and an increase in the control condition that approached significance (*M*_diff_ = 0.24, *p* = 0.069). At Time 1, participants’ mean negative deactivating was significantly lower in the self-compassion condition than in the control condition (*M*_diff_ = −0.47, *p* = 0.036). There was a significant decrease in negative deactivating affect between Time 1 and Time 2 for both control and self-compassion conditions (*M*_diff_ = −0.72, *p* < 0.001; *M*_diff_ = −0.52, *p* < 0.001, respectively). There was no significant difference in negative deactivating affect between conditions at Time 2.

Results for positive activating affect showed a significant main effect of time, *F*(1.94,335.70) = 5.60, *p* = 0.004, $\eta_{\text{p}}^{2}$ = 0.03, whereas the interaction between time and condition was not significant, *F*(1.94,335.70) = 0.90, *p* = 0.41, $\eta_{\text{p}}^{2}$ = 0.01 (see Figure 2C). Positive activating affect did not increase between Time 0 and Time 1 in both conditions. At Time 1, participants’ mean positive activating in the self-compassion condition was higher than in the control condition but not significantly (*M*_diff_ = 0.28, *p* = 0.18). In the control condition there was a significant increase between Time 0 and Time 2 (*M*_diff_ = 0.39, *p* = 0.005) and between Time 1 and Time 2 (*M*_diff_ = 0.36, *p* = 0.002). There was no significant difference in positive activating affect between conditions at Time 2.

For positive deactivating affect we found a significant main effect of time, *F*(2,346) = 23.73, *p* < 0.001, $\eta_{\text{p}}^{2}$ = 0.12, and a significant interaction between time and condition, *F*(2,346) = 3.33, *p* = 0.037, $\eta_{\text{p}}^{2}$ = 0.02 (see Figure 2D). Contract analyses show that there was a significant increase between the Time 0 and Time 1 measure in the self-compassion condition (*M*_diff_ = 0.68, *p* < 0.001), but not in the control condition. At Time 1 participants mean positive deactivating affect was higher in the self-compassion condition than in the control condition (*M*_diff_ = 0.52, *p* = 0.011). Subsequently, positive deactivating affect increased significantly between Time 1 and Time 2 in the control condition (*M*_diff_ = 0.37, *p* = 0.002), but not in the self-compassion condition. Therefore, the difference in positive deactivating affect between the conditions was no longer significant at Time 2.

## Discussion

Job search is necessary to find a job but also negatively impacts job seekers’ feelings due to an abundance of negative job search experiences, leading to reduced well-being. In the current study we tested whether reflecting on negative job search experiences with self-compassion through a writing exercise can alter job seekers’ state self-compassion, and subsequently reduce their negative affect and increase their positive affect in comparison to reflecting on negative job search experiences freely.

Our results extend previous experimental research on self-compassion (Leary et al., 2007; Breines and Chen, 2012). We found that the online self-compassion writing exercise had a small to medium-sized positive impact on job seekers’ state self-compassion in comparison to reflecting freely. Since there were no significant differences between conditions in trait self-compassion before the intervention this finding shows that online writing exercises can effectively induce job seekers’ state self-compassion with regard to their negative job search experiences, suggesting that people’s self-compassion is malleable. Furthermore, we found that state self-compassion related positively to positive affect and negatively to negative affect. As we found no significant direct effects of the intervention on affect, but only through increased state self-compassion, our findings indicate that an increase in self-compassion is the explaining mechanism for our findings on job seekers’ positive and negative affect.

Our findings further show that the effect of the self-compassing writing exercise on affect is partly explained by reduced self-criticism. Specifically, through increasing job seekers’ state self-compassion the intervention negatively influenced their self-criticism, which related positively to negative affect and negatively to positive affect. These findings can be explained by self-compassion theory, which states that self-compassioned individuals are better able to withstand negative experiences as these are not amplified by harsh self-judgment (Neff, 2003a). Previous research has shown a negative relation between self-compassion and self-criticism (Gilbert and Procter, 2006). Our study extends these findings by showing that reduced self-criticism in part mediates the relation between self-compassion and affect.

Even though the indirect effects of the intervention on each of the four types of affect were significant, the repeated measures analyses showed somewhat more support for the intervention effects for deactivating affect than for activating affect. Specifically, after the intervention (Time 1) job seekers reported significantly less negative deactivating affect (e.g., sadness) and more positive deactivating affect (e.g., calmness) as compared to the control condition, while there were no significant differences between the two conditions in positive activating affect (e.g., cheerfulness) and those for negative activating affect (e.g., frustration) only approached significance. We might therefore tentatively conclude that deactivating emotions are somewhat more affected by the self-compassion intervention than activating emotions. This finding supports the theoretical importance of distinguishing between types of affect based on not only the hedonic tone but also their activation level (cf. Feldman Barrett and Russell, 1998; Russell, 2003).

Previous self-compassion intervention studies have looked at various outcomes, such as depressive symptoms and happiness (Shapira and Mongrain, 2010), general negative affect (Leary et al., 2007), improvement motivation (Breines and Chen, 2012), and psychopathological outcomes in clinical setting such as trauma recovery (Zeller et al., 2015) or chronic self-loathing (Krawitz, 2012), but did not include all four types of affect as outcome variables. This study therefore contributes to self-compassion research by generating insight into the impact of self-compassion interventions on affect of different activation levels, that is, by showing differential effects for different types of affect. Our results are thus partly in line with previous research, in that existing research suggests that self-compassion is beneficial for both deactivating and activating affect. For example, Kreemers et al. (2018) showed that trait self-compassion related similarly to affect with different activation levels: negatively to negative (de)activating affect and positively to positive (de)activating affect. This pattern of findings is also apparent in our study between trait self-compassion and affect as measured at Time 0 (see Table 1) and between state self-compassion measured at Time 1 and Time 2 (see Table 1). The fact that we do not seem to find the same effects of the intervention on affect may be attributed to the modesty of the intervention. A relatively short writing exercise filled out once can be expected to have less impact on state self-compassion than a week of daily exercises such as in the study of Shapira and Mongrain (2010). They conducted an intervention study with Canadian adults (Shapira and Mongrain, 2010) with seven daily repeated self-compassion writing exercises. Their intervention reduced depressive symptoms and increased participants’ experience of happiness. The decrease in depressive symptoms after a self-compassion intervention is in line with our results as these symptoms could be categorized as negative deactivating affect. The increase in happiness, however, is not in line with our results, as happiness is a form of positive activating affect and we did not find an increase in positive activating affect after our intervention.

The differences between the intervention and control condition in our study were no longer apparent in the follow-up measurement 1 week after the exercise. In both conditions job seekers felt better: they reported less negative affect and more positive deactivating affect in the Time 2 follow-up in comparison to the baseline. A potential explanation for finding immediate but no long-lasting effects may be the small scope of our self-compassion intervention. Another explanation might be that the experience of writing on one’s worst job search experience aroused negative affect and lowered positive affect, which then was mitigated by the self-compassion intervention. In the control group time seemed to mitigate this negative emotional impact. Nevertheless, job seekers in the self-compassion condition felt better sooner (i.e., immediately after the intervention), while job seekers in the control condition caught up feeling equally well 1 week later. This is an important finding, since job search requires continuous effort over time.

Our findings seem to contrast with those of Shapira and Mongrain (2010), who found that the beneficial effects of a self-compassion intervention only became apparent 3 months after the exercises, even though their intervention was stronger, consisting of seven daily repeated self-compassion writing exercises. However, their outcome measures may be harder to influence as they focused on happiness and depressive symptoms rather than more momentary affect. Combining our results with those of Shapira and Mongrain (2010) we conclude that the effect of a self-compassion intervention on how people feel depends on the frequency with which people are stimulated to be self-compassioned and the malleability of the targeted outcomes. To have a lasting effect on people’s general sense of happiness or dejection, self-compassion likely needs some time to be learned and routinized. These results emphasize the importance of taking time into account when researching the dynamics of emotion regulation through self-compassion by having multiple follow-up measurements.

### Implications

Our findings have implications for both research and practice in the context of job search. Although previous correlational research (Kreemers et al., 2018) demonstrated that trait-self-compassion beneficially relates to job seekers’ affect, it could not ascertain causal relationships. In the current study, we address the call that various scholars (e.g., Aguinis and Edwards, 2014; Eden, 2017) made to adopt experimental field designs to provide more rigorous tests of our theories. By adopting an experimental design our results support the causal effects of self-compassion on job seekers’ emotional responses, which provides evidence for self-compassion theory in the context of job seeking. In addition, we contribute to our understanding of how self-compassion makes job seekers feel better by identifying self-criticism as mediator in the relation between self-compassion and affect. Hereby we add weight to the notion that self-compassion may help to reduce self-criticism and that interventions could be tailored to specific sensitive groups (e.g., those who suffer more from self-criticism; Shapira and Mongrain, 2010).

Furthermore, by showing that self-compassion has the potential to aid job seekers in effectively dealing with negative job search experiences, we gained insights that can be implemented in practice (e.g., unemployment counseling; career counseling; and job search interventions). The writing exercises can function as a starting point to further develop or extend interventions aimed at job seekers taking a self-compassioned perspective toward experienced difficulties, which can help them to retain or increase the positive emotions that benefit their job search. Thus far, stress management interventions have not been found effective in the context of job search (see Liu et al., 2014 for a meta-analysis). Self-compassion may not only benefit job seekers’ emotional well-being but may also improve their motivation, as was shown in prior research on people’s responses to negative feedback (Breines and Chen, 2012). Considering that job seekers have to continue to search for a job also after negative feedback, self-compassion could encourage them to do so. In future research job search success should therefore also be taken into account as an outcome of a self-compassion intervention.

Our results add to the growing body of literature that distinguishes different activation levels of affect (e.g., Watson and Tellegen, 1985; Taylor, 1991; Feldman Barrett and Russell, 1998; Russell, 2003; Carver, 2004; Baas et al., 2008; Yik et al., 2011; Wrzus et al., 2015) by showing that our self-compassion intervention was effective for positive and negative deactivating affect but less so for activating affect. In other words, reflecting on negative job search experiences generally made job seekers calmer and less sad but not more cheerful or less frustrated than reflecting freely. These outcomes are perhaps explainable by self-compassion’s self-soothing properties. According to the social mentality theory (Gilbert, 1989), which draws on principles of evolutionary biology, neurobiology, and attachment theory, self-compassion deactivates the threat system (associated with feelings of insecurity, defensiveness and the limbic system) and activates the self-soothing system (associated with feelings of secure attachment, safeness, and the oxytocin–opiate system). This self-soothing system speaks more to deactivating affect and might therefore explain why a self-compassion intervention influences these emotions more strongly.

### Limitations and Future Research

To put our results into perspective, we have to take the limitations of our research into account. First, our intervention was small and its effects were modest. Our results show that a self-compassion exercise has a small beneficial impact on state self-compassion, self-criticism, and affective responses to negative job search experiences immediately after the intervention. Our results also show that the impact of the exercises on affect were matched by the control condition after a week. Given that our intervention was small and performed in a field setting, which carries more noise than a laboratory setting, having a modest effect is understandable and even promising (Prentice and Miller, 1992). In recent years there has been a development toward briefer and more precise psychological interventions, called wise interventions (Walton, 2014). These interventions aim to “alter a specific way in which people think or feel in the normal course of their lives to help them flourish” (Walton, 2014, p. 73) and they are furthermore characterized by lasting effects over time. Inducing self-compassion fits that description of a wise intervention. However, wise interventions will affect long-term outcomes only if they alter critical recursive processes. Future research could therefore focus on ways to make a self-compassion intervention more recursive.

Second, several issues should be taken into account regarding the generalizability of our findings. Our sample was rather small, relatively highly educated, and included mostly women. Future research is needed to develop and test a self-compassion intervention targeted to lower-educated job seekers. Also, because women generally have somewhat lower levels of self-compassion (Yarnell et al., 2015), a self-compassion intervention may be more useful for women than for men. Our sample size was too small for detecting such interaction effects, but future research should examine to what extent a self-compassion intervention has differential effects for men and women. Further, we tested the effectiveness of our self-compassion intervention in a mixed sample of job seekers, including new entrants, employed, and unemployed job seekers. Although job search is relevant in all these groups (e.g., Kanfer et al., 2001; Boswell et al., 2012; Van Hooft et al., in press), future research is needed to examine to what extent our findings generalize to especially vulnerable populations such as long-term unemployed individuals. Possibly, stronger interventions are needed in such groups to obtain lasting effects.

Third, this study presents a first step to testing the effectiveness of a self-compassion intervention for job seekers. Although we showed that the intervention impacted job seekers’ affective responses beneficially, we need additional data to test whether the improved affective responses can translate to better job search cognitions, behaviors, and outcomes. Future research should therefore examine whether self-compassion affects relevant job search cognitions and behaviors such as job search strategies (Crossley and Highhouse, 2005), metacognitive activities (Turban et al., 2009), job search intensity (Blau, 1994), job search quality (Van Hooft et al., 2013), and job search success (e.g., interview invitations and job offers).

Fourth, our mediators and outcomes were assessed using self-report measures. Although it can be argued that our focal constructs such as state self-compassion and self-criticism can best be measured through self-reports, future research may examine whether a self-compassion intervention has visible effects on affect as perceived by others and on more objective outcomes such as job attainment. Further, similar to previous research (e.g., Breines and Chen, 2012), we measured state self-compassion with a relatively brief scale. Therefore, we could not examine which self-compassion dimension(s) carried the effects of the intervention. Future research should develop multiple-item state self-compassion scales for each of the three self-compassion dimensions and test the effects for the dimensions separately.

Last, all participants were asked to reflect on their worse negative job search experience. This reflection constitutes a wide range of experiences that happened to the job seekers longer or less long ago. We did not control for the intensity of the experience or the time that had been passed since the event had happened, as we were mostly interested in the difference between the conditions. Moreover, job seekers were randomly divided over the conditions. Still, future research could explore how different experiences can contribute to different emotional responses.

## Conclusion

Our results show that a self-compassion intervention consisting of a writing exercise promotes state self-compassion and has a beneficial short-term effect on job seekers’ deactivating emotions. Moreover, state self-compassion reduces self-criticism among job seekers, which is beneficial for their affective responses to negative job search experiences. These results can inspire the development of self-compassion interventions and can be implemented in practice (e.g., unemployment counseling, career counseling, and job search interventions).

## Data Availability Statement

The datasets generated for this study are available on request to the corresponding author.

## Ethics Statement

The studies involving human participants were reviewed and approved by Faculty Ethics Review Board (ERB) of the Faculty of Social and Behavioural Sciences of the University of Amsterdam. The patients/participants provided their online informed consent to participate in this study.

## Author Contributions

LK, EH, AV, and SS contributed to the conception and design of the study. LK and SS organized the data collection. LK, EH, and SS performed the statistical analysis. LK, EH, and AV contributed to writing the manuscript. All authors contributed to the article and approved the submitted version.

## Conflict of Interest

The authors declare that the research was conducted in the absence of any commercial or financial relationships that could be construed as a potential conflict of interest.

## Footnotes

1 Due to logistic reasons the time between the baseline questionnaire and the intervention varied. The average time was 6.81 days (*SD* = 2.71). These varying times are not problematic as the average time between Time 0 and Time 1 did not differ between conditions, *t*(178) = 1.22, *p* = 0.225, and our research questions focus on differences between the conditions.

2 In addition to the variables mentioned in the Section “Materials and Methods,” the Time 0 questionnaire also contained various other personality traits (i.e., perfectionism, self-esteem, learning goal orientation, openness to change, incremental beliefs regarding emotions, and neuroticism), potential mediators (i.e., fear of failure, job search self-efficacy, and rumination), and dependent variables (i.e., exhaustion, vitality, job search intention, and job search time). At Time 1 participants were first asked whether they had negative job search experiences and to what extent they perceived job search progress in the last month before proceeding with the writing task. After the intervention the same mediator and dependent variables were measured as in the Time 0 questionnaire and these were also measured at Time 2.

3 The negative job search experiences that participants described, included experiences related to forethought and exploration (e.g., realization that one’s educational or work profile does not fit the qualifications necessary for the type of job people are interested in), preparatory job search behavior (e.g., not meeting advertised job requirements due to lack of work experience; negative networking experiences), active job search behavior (e.g., self-doubt in how to present oneself; not being taken seriously or negative interactions in the job interview), selection result (e.g., unfair or unexpected rejections; poor communication of the rejection), and the entire job search process (e.g., insecurity of finding a job; dissatisfaction with progress and feeling stuck; indifference of hiring organizations). For more information, see Kreemers (2020).

4 For the pilot test we asked seven individuals to complete the writing exercise in presence of a researcher, using a think-aloud instruction. Based on the pilot we slightly rephrased the third question by adding a more specific instruction (i.e., “Imagine your emotions as clouds drifting by. Which emotions are drifting by? Please describe them.”).

5 We originally selected the same four items as Breines and Chen (2012). In adjusting the items to the job search context one item became conceptually illogical as part of a state self-compassion measure right after the writing exercise, because it addressed job seekers’ self-compassioned attitudes during job search, while there had been no time to search since the exercise. Therefore, we decided to run the analyses with the remaining three items which each represented one of the three components of self-compassion.
